# Association of Levels of Physical Activity With Risk of Parkinson Disease: A Systematic Review and Meta-analysis

**DOI:** 10.1001/jamanetworkopen.2018.2421

**Published:** 2018-09-21

**Authors:** Xuexian Fang, Dan Han, Qi Cheng, Pan Zhang, Chunhui Zhao, Junxia Min, Fudi Wang

**Affiliations:** 1 School of Public Health, The First Affiliated Hospital, Institute of Translational Medicine, Zhejiang University School of Medicine, Hangzhou, China; 2 Precision Nutrition Innovation Center, School of Public Health, Zhengzhou University, Zhengzhou, China; 3 State Key Laboratory of Industrial Control Technology, College of Control Science and Engineering, Zhejiang University, Hangzhou, China

## Abstract

**Importance:**

The association between physical activity and the risk of Parkinson disease (PD) has been increasingly recognized. However, to our knowledge, a comprehensive assessment of the quantitative dose-response association between physical activity and PD risk has not been performed previously.

**Objective:**

To quantify the dose-response association between physical activity and the risk of developing PD.

**Data Sources:**

PubMed, Embase, and Web of Science were systematically searched for peer-reviewed articles published through February 2018 reporting the association between physical activity and PD risk.

**Study Selection:**

Prospective studies that examined the association between physical activity and PD risk were included.

**Data Extraction and Synthesis:**

Data were extracted independently by 2 investigators. Adjusted risk estimates were extracted and pooled using a random-effects model. The study adhered to Meta-analysis of Observational Studies in Epidemiology (MOOSE) reporting guidelines.

**Main Outcomes and Measures:**

The main outcome was the incidence of PD diagnoses confirmed by the treating neurologists or the authoritative medical records.

**Results:**

Eight prospective studies totaling 544 336 participants included 2192 patients with PD with a median (range) follow-up period of 12 (6.1-22.0) years were identified. A significantly reduced risk of PD was associated with the highest levels of either total physical activity (relative risk, 0.79; 95% CI, 0.68-0.91) or moderate to vigorous physical activity (relative risk, 0.71; 95% CI, 0.58-0.87), with stronger associations among men than among women. In contrast, light physical activity was not associated with PD risk (relative risk, 0.86; 95% CI, 0.60-1.23). The dose-response analysis revealed that for each 10 metabolic equivalent of task–hours/week increase in total or moderate to vigorous physical activity, the risk of PD among men decreased by 10% and 17%, respectively. No linear dose-response association was found between physical activity and PD risk among women.

**Conclusions and Relevance:**

This analysis revealed an inverse dose-response association between physical activity and PD risk among men; importantly, even moderate exercise was associated with a significant reduction in the risk of PD. Future studies with quantified measurements of physical activity will help identify the precise relative risk estimates for various levels of activity with respect to PD risk.

## Key Points

**Question:**

What is the association between physical activity and the risk of Parkinson disease?

**Findings:**

In this systematic review and meta-analysis of more than half a million unique participants, physical activity, particularly moderate to vigorous physical activity, was associated with a significant reduction in Parkinson disease risk. This association was stronger among men than women.

**Meaning:**

Physical activity may be an important protective factor for preventing the development of Parkinson disease among at-risk men; thus, large prospective studies should be performed to examine this association and to investigate the factors that underlie the observed sex difference.

## Teaser

This systematic review and meta-analysis investigates the dose-response association between levels of physical activity and the risk of Parkinson disease by examining data from 8 studies including more than half a million participants.

## Introduction

Parkinson disease (PD) is an aging-related neurodegenerative disorder characterized by progressive motor impairment.^1^ It is the second most common neurodegenerative disease (after Alzheimer disease), affecting more than 1% of people aged 65 years and older.^2,3^ In the United States, the incidence of parkinsonism and PD increased considerably between 1976 and 2005, particularly among men 70 years of age and older.^4^ Moreover, a cross-sectional survey revealed that an estimated 1.7 million people aged 55 years and older in China have PD.^5^

The etiology of PD is poorly understood but likely involves both genetic and environmental factors.^6^ In the past 2 decades, the results of a series of prospective cohort studies suggested that lifestyle factors likely modify the risk of developing PD.^7^ One such factor, physical activity, has long been known to reduce the risk of a wide range of diseases and conditions, including cardiovascular disease, stroke, and diabetes.^8,9,10^

Recently, a growing body of evidence has suggested that increased physical activity may also reduce the risk of PD.^11^ However, these studies varied with respect to sample size, ethnicity, and other characteristics, thereby leading to inconsistencies with respect to their interpretation. In addition, relatively few studies systematically quantified the putative dose-response relationship between physical activity and PD risk. Therefore, we performed a dose-response meta-analysis of published prospective studies to obtain quantitative estimates of the association between physical activity and PD risk.

## Methods

### Search Strategy

We followed the Meta-analysis of Observational Studies in Epidemiology (MOOSE) reporting guideline.^12^ A systematic literature search for prospective studies up to February 28, 2018, was conducted in the databases, including PubMed, Embase, and Web of Science, using the following keywords for the literature search: (“physical activity” OR “motor activity” OR “exercise”) AND (“Parkinson disease” OR “Parkinson’s disease” OR “Parkinsons disease”). The search had no language restriction. We also searched and reviewed the references cited within the retrieved relevant reports for any additional studies.

### Study Selection

Studies were included in the current meta-analysis if they met the following 4 criteria: (1) they used prospective study design, including cohort and nested case-control studies; (2) the exposure of interest was physical activity; (3) the outcome was the incidence of PD; and (4) the authors reported the risk estimate with 95% confidence intervals. We excluded retrospective studies, studies in animals, nonoriginal research (reviews, editorials, or commentaries), abstracts, and duplicated studies. To ensure the correct identification of eligible studies, we used a 2-step selection process. First, 2 investigators (X.F. and D.H.) independently conducted the literature search and initial screening of all titles and abstracts; the full text of each potentially relevant article was then evaluated. Discrepancies were resolved through group discussion with a third investigator (F.W.).

### Data Extraction

A standardized data collection form was used to extract data. From each retrieved study, we extracted the following information: the first author’s name, year of publication, country where the study was conducted, name of the study (where applicable), study design (cohort or nested case-control study), participant age at baseline, duration of follow-up, participant sex, sample size (ie, the number of cases and/or participants), quintiles of baseline physical activity, and corresponding risk estimates of PD with 95% confidence intervals. For studies that did not categorize physical activity qualitatively, most fully adjusted risk estimate for the highest compared with the lowest quintile of physical activity and the corresponding 95% confidence interval was recorded. Two independent investigators (X.F. and D.H.) performed the data extraction process, and any disagreements were resolved by group discussion.

The quality of included studies was evaluated according to the Newcastle-Ottawa scale for nonrandomized studies. A maximum of 9 points was assigned to each study as follows: 4 for the selection of participants and the measurement of exposure, 2 for comparability, and 3 for the assessment of outcomes and adequate follow-up. A score of 0 to 3, 4 to 6, or 7 to 9 was regarded as low-, moderate-, or high-quality, respectively.

### Statistical Analysis

In this meta-analysis, the relative risks (RRs) with 95% confidence intervals were considered as the common measure of associations across studies; where necessary, the hazard ratio and/or incidence rate ratio were used to approximate RRs. For the comparison between the highest and lowest categories of physical activity, we calculated the summarized RRs and their corresponding 95% confidence intervals using a random-effects model, which could incorporate both within- and between-study variability.

Owing to the relatively wide range of definitions for categories of physical activity in the included studies, a dose-response analysis based on an increase in physical activity of 10 metabolic equivalent of task–hours (MET-hours) per week was conducted by using the method described by Greenland and Longnecker^13^ and the publicly available Stata statistical software code written by Orsini and colleagues.^14^ According to the method, we extracted the categories of physical activity, the distributions of cases and person-years, and RRs with 95% confidence intervals. If the number of cases or person-years was not available, variance-weighted least-squares regression was performed to calculate the summarized risk estimate.^15^ The median or mean value in each category was used as the corresponding dose of physical activity. If neither median nor mean value was reported, we considered the midpoint of the upper and lower boundaries as the dose of each category. If the highest and/or lowest category was open ended, the midpoint of that category was set by assuming that the categorical width was the same as the next adjacent category. To evaluate a potential curvilinear association between physical activity and PD risk, we conducted a restricted cubic spline model with 3 knots at the 10th, 50th, and 90th percentiles of the distribution.^16^

Heterogeneity among studies was estimated by the *I^2^* statistic,^17^ and we considered the values of low, moderate, and high *I^2^* metric to be 25%, 50%, and 75%, respectively. To examine the significance of the difference in RRs and the possible influence of residual confounding factors, we performed subgroup analyses on possible sources of heterogeneity, including sex, geographic location, follow-up, sample size, and study quality.

We assessed the potential for publication bias using Egger linear regression tests and Begg rank correlation tests. All statistical analyses were performed using Stata statistical software version 12 (StataCorp), and all *P* values were 2-sided with a significance level of .05.

## Results

### Literature Search and Study Characteristics

The study selection process and the results of the literature search are depicted in eFigure 1 in the Supplement. Using our search strategy, we identified 5088 articles in PubMed, 4978 articles in Embase, and 2907 articles in Web of Science. After duplicate articles were removed, 5274 articles remained; 5249 of these articles were excluded based on the title and/or abstract, leaving 25 potentially relevant articles for full-text review. After applying further exclusion criteria, a total of 8 prospective studies (published in 7 articles) were included in our analysis,^18,19,20,21,22,23,24^ including 544 336 participants and 2192 patients with PD with a median (range) follow-up of 12 (6.1-22.0) years. Among these studies, the report by Sasco and colleagues,^21^ a nested case-control study, is the first epidemiologic investigation of the effect of physical activity on the etiology of PD. We included the study because this normative study met the inclusion criteria of our meta-analysis.

The characteristics of these 8 studies are summarized in the Table. Six studies^18,19,21,22,23^ were conducted in the United States, 1 study^20^ in Finland, and 1 study^24^ in Sweden. All studies used self-reported physical activity, which was collected using questionnaires or interviews. The study quality scores ranged from 6 to 9, with a mean (SD) score of 7.9 (1.1) (Table; eTable 1 in the Supplement).

**Table.** Characteristics of Included Studies

Source	Location	Study Name	Design	Sex	Age, y	Follow-up, y	Cases, No./Participants, No.	Quality^a^
Chen et al,^18^ 2005	United States	Health Professionals Follow-up Study	Cohort	Male	40-75	14	252/48 574	9
		Nurses’ Health Study		Female	30-55	12	135/77 254	
Logroscino et al,^19^ 2006	United States	Harvard Alumni Health Study	Cohort	Female	67.6 (8.1)^b^	6.1	101/10 714	8
Sääksjärvi et al,^20^ 2014	Finland	Finnish Mobile Clinic Health Examination Survey	Cohort	Both	50-79	22	101/6715	7
Sasco et al,^21^ 1992	United States	NA	Nested case-control	Male	NA	NA	137/685	6
Thacker et al,^22^ 2008	United States	CPS II Nutrition Cohort	Cohort	Both	63^c^	9	413/143 325	8
Xu et al,^23^ 2010	United States	NIH-AARP Diet and Health Study	Cohort	Both	50-71	10	767/213 701	9
Yang et al,^24^ 2015	Sweden	Swedish National March Cohort	Cohort	Both	50.3 (17.1)^b^	12.6	286/43 368	8

Abbreviations: AARP, American Association of Retired Persons; CPS, Cancer Prevention Study; NA, not available; NIH, National Institutes of Health.

^a^ Quality assessment was performed using the Newcastle-Ottawa scale. Scores of 0 to 3, 4 to 6, and 7 to 9 are regarded as low, moderate, and high quality, respectively.

^b^ The mean (SD) age of participants at baseline was reported.

^c^ The mean age of participants at baseline was reported.

### Categorical Association Between Physical Activity and PD Risk

**Figure 1.**
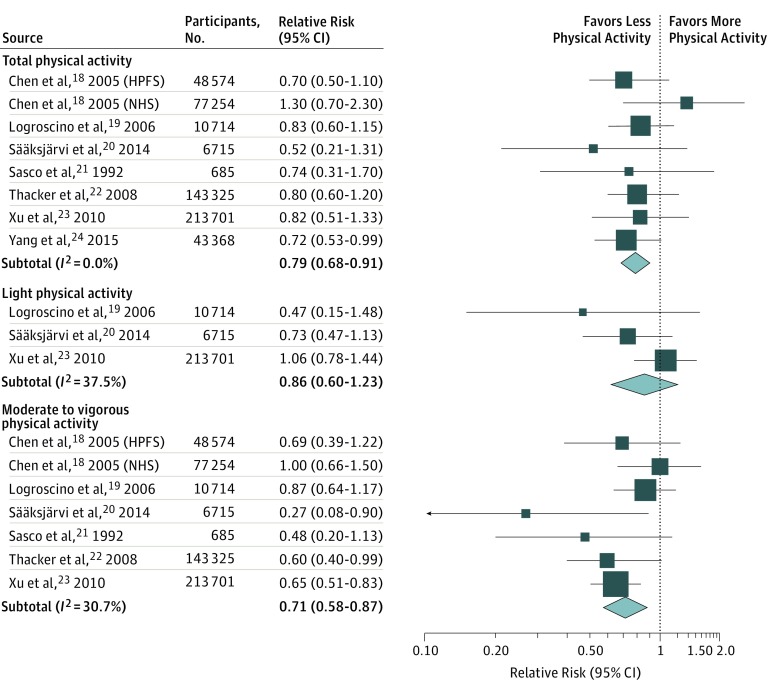
Parkinson Disease Risk for Highest vs Lowest Categories of Physical Activity The size of each box indicates the study’s weight in the analysis. HPFS indicates Health Professionals Follow-up Study; NHS, Nurses’ Health Study.

The multivariable-adjusted RRs of PD for the highest vs the lowest category of physical activity in each study, and for all studies combined, are shown in Figure 1. Among the 8 studies, only 1 found a statistically significant inverse correlation between physical activity and PD risk; however, the pooled RR of PD was 0.79 (95% CI, 0.68-0.91) when we compared the highest vs the lowest category of physical activity. No heterogeneity was observed across the studies (*I^2^* = 0%).

This association was due entirely to moderate to vigorous activity. Specifically, participants in this highest category of activity had a 29% lower risk of PD than those who reported no moderate to vigorous activity (RR, 0.71; 95% CI, 0.58-0.87; *I^2^* = 30.7%). In contrast, light physical activity was not significantly correlated with PD risk (RR, 0.86; 95% CI, 0.60-1.23; *I^2^* = 37.5%).

Considering the possibility of the reverse causation between early PD with decreased physical activity, we conducted a time-lag meta-analysis using 6 studies excluding the first 4 to 10 years of follow-up.^18,20,22,23,24^ The results of the time-lag analysis were similar to our major findings presented in this study, suggesting that such reverse causality is unlikely (eFigure 2 in the Supplement).

### Subgroup Analyses

**Figure 2.**
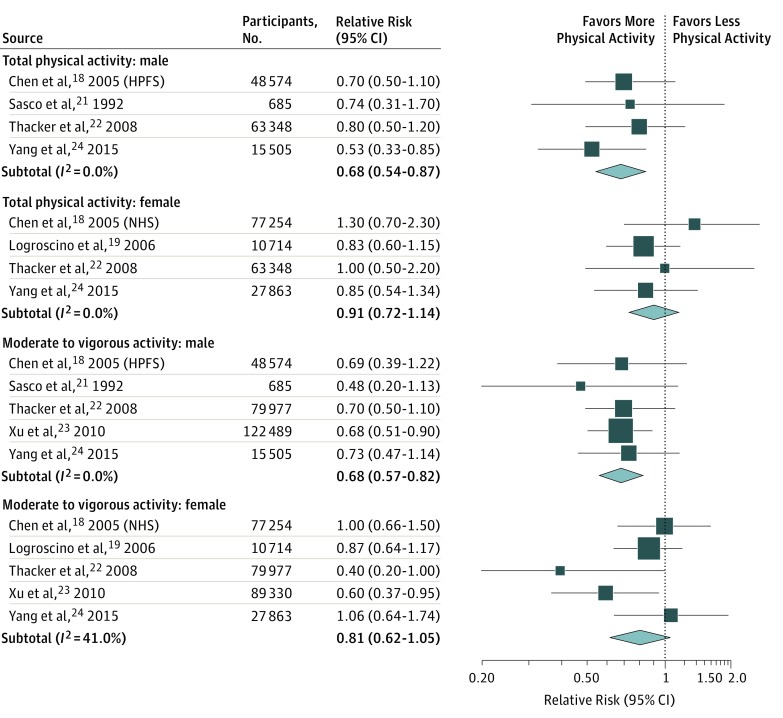
Parkinson Disease Risk by Sex for the Highest vs the Lowest Categories of Total and Moderate to Vigorous Physical Activity HPFS indicates Health Professionals Follow-up Study; NHS, Nurses’ Health Study.

The results of our subgroup analyses stratified by study design and study population are summarized in eTable 2 in the Supplement. Our analysis revealed that the association between physical activity and the risk of PD was not substantially changed by geographic region, follow-up duration, population size, or study quality. Notably, however, the association between physical activity and PD risk was more robust among men, regardless of whether we examined total physical activity (RR, 0.68; 95% CI, 0.54-0.87) or moderate to vigorous activity (RR, 0.68; 95% CI, 0.57-0.82), with little heterogeneity (Figure 2).

### Linear Dose-Response Association Between Physical Activity and PD Risk

**Figure 3.**
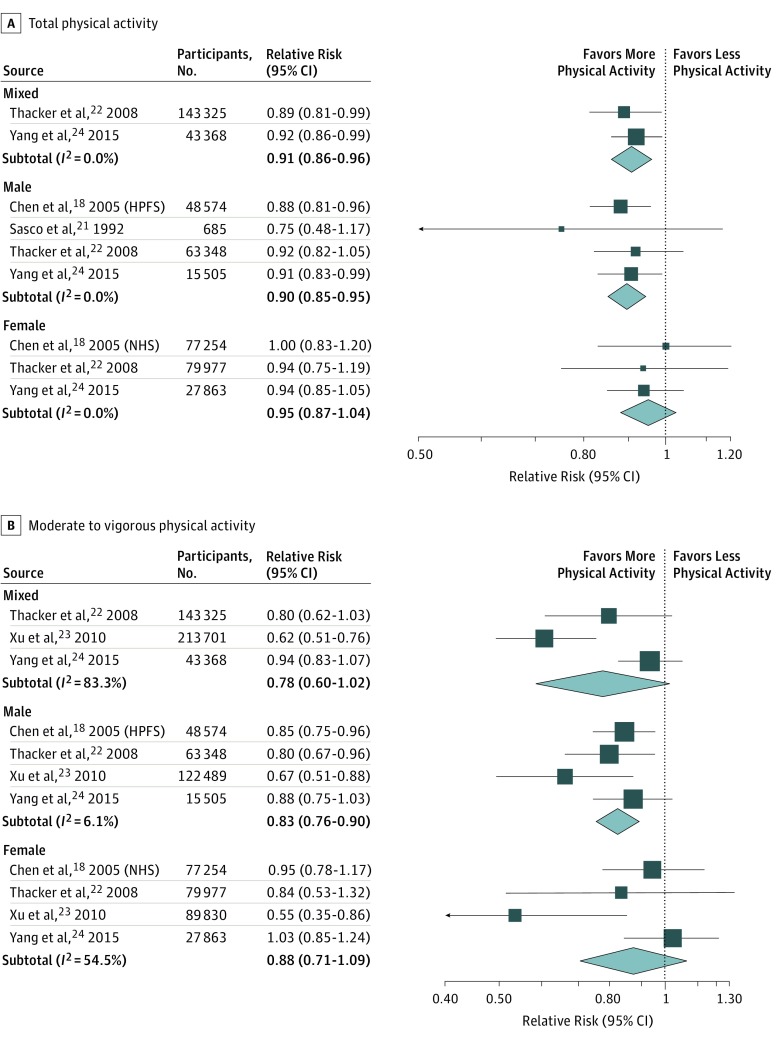
Parkinson Disease Risk per 10 Metabolic Equivalent of Task–Hour Increase The plot shows sex-specific linear relative risk for each 10-hour increase in metabolic equivalent of task–hours per week for total physical activity (A) and moderate to vigorous physical activity (B). HPFS indicates Health Professionals Follow-up Study; NHS, Nurses’ Health Study.

After we excluded 2 studies due to a lack of detailed physical activity categories,^19,20^ we examined the sex-specific linear RR (and 95% confidence intervals) for increase of 10 MET-hours/week, sorted by category (Figure 3A). This dose-response analysis revealed that each increase of 10 MET-hours/week in total physical activity decreased the risk of PD by 10% in men (RR, 0.90; 95% CI, 0.85-0.95; *I^2^* = 0%) and 9% in mixed-sex populations (RR, 0.91; 95% CI, 0.86-0.96; *I^2^* = 0%). In contrast, we found no linear dose-response relationship between total physical activity and PD risk among women (RR, 0.95; 95% CI, 0.87-1.04; *I^2^* = 0%) (Figure 3A).

With respect to the effects of moderate to vigorous activity, the reduced risk of PD was observed only in men, and not in either the female or mixed-sex populations (Figure 3B). Specifically, among the male participants the pooled RR of PD associated with a 10 MET-hours/week increase in moderate to vigorous activity was 0.83 (95% CI, 0.76-0.90) with low heterogeneity (*I^2^* = 6.1%).

### Continuous Dose-Response Association Between Physical Activity and PD Risk

**Figure 4.**
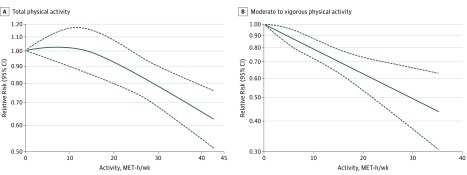
Dose-Response Analyses Dose-response analyses of the nonlinear association between total (A) and moderate to vigorous (B) physical activity and the risk of Parkinson disease. The solid line represents point estimates of association between physical activity and Parkinson disease risk; the dashed lines indicate 95% confidence intervals. MET indicates metabolic equivalent of task.

Figure 4 depicts the continuous dose-response association between quantitative estimates of physical activity (MET-hours per week) and PD risk. Higher levels of either total (Figure 4A) or moderate to vigorous (Figure 4B) physical activity were consistently associated with a lower risk of PD.

### Publication Bias

Begg rank correlation and Egger linear regression tests revealed little evidence of publication bias with respect to physical activity in relation to PD risk.

## Discussion

To our knowledge, this meta-analysis is the largest and most comprehensive evaluation of the dose-response relationship between physical activity and the risk of PD in the general population. Using data extracted from prospective studies, our pooled analysis of more than half a million adults revealed that higher levels of physical activity—particularly moderate to vigorous activity—are associated with a lower risk of developing PD. This association remained when we performed subgroup analyses based on geographical region, follow-up duration, sample size, and study quality. Importantly, however, the beneficial effect of physical activity on the risk of PD was exclusive to men and was not observed among the women in the studies.

In 1992, Sasco and colleagues^21^ first suggested that increased physical activity may have a protective effect against PD, reporting that men who played sports in college and/or in adulthood have a decreased risk of developing PD; moreover, they found that higher levels of physical activity were associated with a progressively lower risk of PD. Since this initial report, a series of subsequent epidemiological studies investigated this putative relationship, yielding compelling results. For example, higher levels of exercise were found to reduce the risk of PD in men in the Health Professionals Follow-up Study but not in women in the Nurses’ Health Study, indicating that men and women may have different biological responses to physical activity.^18^ On the other hand, a previous meta-analysis conducted by Xu et al^23^ found no sex-based difference between physical activity and PD risk. However, these authors compared only the highest activity level with the lowest activity level, and many more cohort studies have been published since their meta-analysis.

Tanaka et al^25^ also measured the effects of a multimodal physical exercise program in 20 elderly patients with PD and found that patients who underwent general physical training for 6 months showed an improvement in executive functioning. Recently, Schenkman et al^26^ suggested that high-intensity exercise on a treadmill may be both feasible and safe for patients with PD. Nevertheless, these promising exercise-induced results should be investigated further in large trials involving patients with PD.

Several mechanisms have been suggested for the putative neuroprotective effect of physical activity. For example, physical activity in animal models of PD has been shown to (1) upregulate the production of various growth factors and receptors, (2) maintain dopaminergic function, and (3) reduce cellular inflammation and oxidative stress.^11,27^ In healthy humans, exercise promotes the expression of neuroprotective growth factors such as brain-derived neurotrophic factor and glial-derived neurotrophic factor.^28,29^ Physical activity may also reduce damage to dopaminergic neurons within motor circuits.^30^ Finally, rodent models of lesion-induced PD have preserved striatal dopamine levels following treadmill activity and have an increased loss of dopaminergic neurons following forced nonuse of the contralateral forelimb.^31,32^

The strength of our meta-analysis lies in 4 key aspects. First, we included all available prospective studies with high quality, large sample size, and sufficiently long-term follow-up data. Second, in addition to performing a traditional categorical meta-analysis, we were also able to quantify—and therefore categorize—the amount of physical activity and assess the risk of PD associated with specific, quantitative levels of physical activity, thereby obtaining more meaningful information. Third, we found no significant heterogeneity across the studies included in our meta-analysis. Fourth, we performed several subgroup analyses and observed significant sex-based differences with respect to the association between physical activity, including exercise intensity, and PD risk.

### Limitations

Our study has several limitations that may affect the interpretation of our results. First, a limited amount of empirical data was appropriate for inclusion in our meta-analysis. Second, residual confounding factors are possible, given that the level of adjustment differed for each study; therefore, we used risk estimates derived from fully adjusted models for our pooled analysis to reduce potential confounding factors. Third, although searching both Embase and PubMed is expected to cover approximately 97.5% of relevant published articles,^33^ and although we also included Web of Science in our search, we cannot exclude the possibility that additional relevant articles may have been missed as a result of restricting our search to these 3 databases. However, in addition to searching these databases, we also manually searched the reference lists of all relevant articles; therefore, we believe that the number of articles missing from our analysis is likely small and would have little impact on our analysis. Fourth, most of the studies included in our analysis collected their information via self-reporting questionnaires, which could have led to errors in the measurement of physical activity. Fifth, we could not entirely rule out the possibility that preclinical or undiagnosed PD pathogenesis at baseline may manifest as a lower level of physical activity. However, a previous study using repeated measurements reported no significant decrease in physical activity level among patients with PD until approximately 2 to 4 years prior to disease diagnosis.^18^ Based on our time-lag meta-analysis, the potential reverse causality between early PD and decreased physical activity should not affect the major findings of this study. Even so, if the presyndromal period is longer than anticipated, even excluding the first 4 to 10 years would not solve the problem.

## Conclusions

We report that higher levels of total physical activity—particularly moderate to vigorous activity—are associated with a reduced risk of PD. These benefits were significant among men, but were less robust among women; specifically, an increase of 10 MET-hours/week in total and moderate to vigorous physical activity decreased the risk of PD risk in men by 10% and 17%, respectively. These findings may help guide physicians and health care policy makers in making recommendations and developing guidelines with respect to the degree of physical activity that can help reduce the risk of PD at both the individual level and the population level. More epidemiological studies with large sample size and detailed quantification of physical activity will help establish more precise information regarding this association.

## Notes

Supplement. **eTable 1.** Quality Assessment of Included Prospective Studies **eTable 2.** Subgroup Analyses of Total Physical Activity and risk of Parkinson Disease (Highest Versus Lowest Category) **eFigure 1.** Flowchart of Literature Search and Selection Strategy **eFigure 2.** Forest Plot of PD Risk for the Highest Versus the Lowest Categories of Physical Activity–Excluding the First 4-10 Years of Follow-Up
