# Supplementary material for: Association of Levels of Physical Activity With Risk of Parkinson Disease: A Systematic Review and Meta-analysis
Source: JAMA Netw Open. 2018 Sep 21;1(5):e182421. doi: 10.1001/jamanetworkopen.2018.2421 (PMC6324511; doi:10.1001/jamanetworkopen.2018.2421)
Supplement: Supplement. — eTable 1. Quality Assessment of Included Prospective Studies eTable 2. Subgroup Analyses of Total Physical Activity and risk of Parkinson Disease (Highest Versus Lowest Category) eFigure 1. Flowchart of Literature Search and Selection Strategy eFigure 2. Forest Plot of PD Risk for the Highest Versus the Lowest Categories of Physical Activity–Excluding the First 4-10 Years of Follow-Up [file jamanetwopen-1-e182421-s001.pdf]

Supplementary Online Content

Fang X, Han D, Cheng Q, et al. Association of Levels of Physical Activity With Risk of Parkinson Disease: A Systematic Review and Meta-analysis. *JAMA Netw Open.* 2018;1(5):e182421. doi:10.1001/jamanetworkopen.2018.2421

eTable 1. Quality Assessment of Included Prospective Studies

eTable 2. Subgroup Analyses of Total Physical Activity and risk of Parkinson Disease (Highest Versus Lowest Category)

eFigure 1. Flowchart of Literature Search and Selection Strategy

eFigure 2. Forest Plot of PD Risk for the Highest Versus the Lowest Categories of Physical Activity–Excluding the First 4-10 Years of Follow-Up

This supplementary material has been provided by the authors to give readers additional information about their work.

© 2018 Fang X et al. *JAMA Network Open.*

eTable 1. Quality assessment of included prospective studies

**Author, year**	**Selection**				**Comparability**	**Outcome**			**Overall quality**
	**Representative of cases**	**Selection of controls**	**Exposure ascertainment**	**No history of disease**	**Comparable on confounders**	**Outcome assessment**	**Adequate follow-up time (> 5 years)**	**Follow-up rate (> 80%)**	
Chen et al, 2005	1	1	1	1	2	1	1	1	9
Logroscino et al, 2006	1	1	1	0	2	1	1	1	8
Sääksjärvi et al, 2014	1	1	1	0	1	1	1	1	7
Sasco et al, 1992	1	0	1	1	1	1	0	1	6
Thacker et al, 2008	1	1	1	1	1	1	1	1	8
Xu et al, 2010	1	1	1	1	2	1	1	1	9
Yang et al, 2015	1	1	1	1	1	1	1	1	8

Average: 7.86

© 2018 Fang X et al. *JAMA Network Open.*

eTable 2. Subgroup analyses of total physical activity and risk of Parkinson disease (highest versus lowest category)

	No. of studies	RR (95% CI)	I^2 (%)
**Gender**			
Male	4	0.68 (0.56-0.82)	0.0
Female	5	0.84 (0.68-1.04)	5.8
**Location**			
US	6	0.82 (0.69-0.98)	0.0
Europe	2	0.70 (0.52-0.94)	0.0
**Follow-up**			
>10 years	4	0.77 (0.59-1.01)	26.3
≤10 years	3	0.82 (0.66-1.01)	0.0
**Participants**			
>50,000	4	0.74 (0.61-0.90)	0.0
≤50,000	4	0.87 (0.68-1.11)	0.0
**Study quality**			
Score>8	3	0.86 (0.61-1.20)	31.1
Score≤8	5	0.77 (0.64-0.92)	0.0

Abbreviations: CI, confidence interval; RR, relative risk.

© 2018 Fang X et al. *JAMA Network Open.*

eFigure 1. Flowchart of literature search and selection strategy

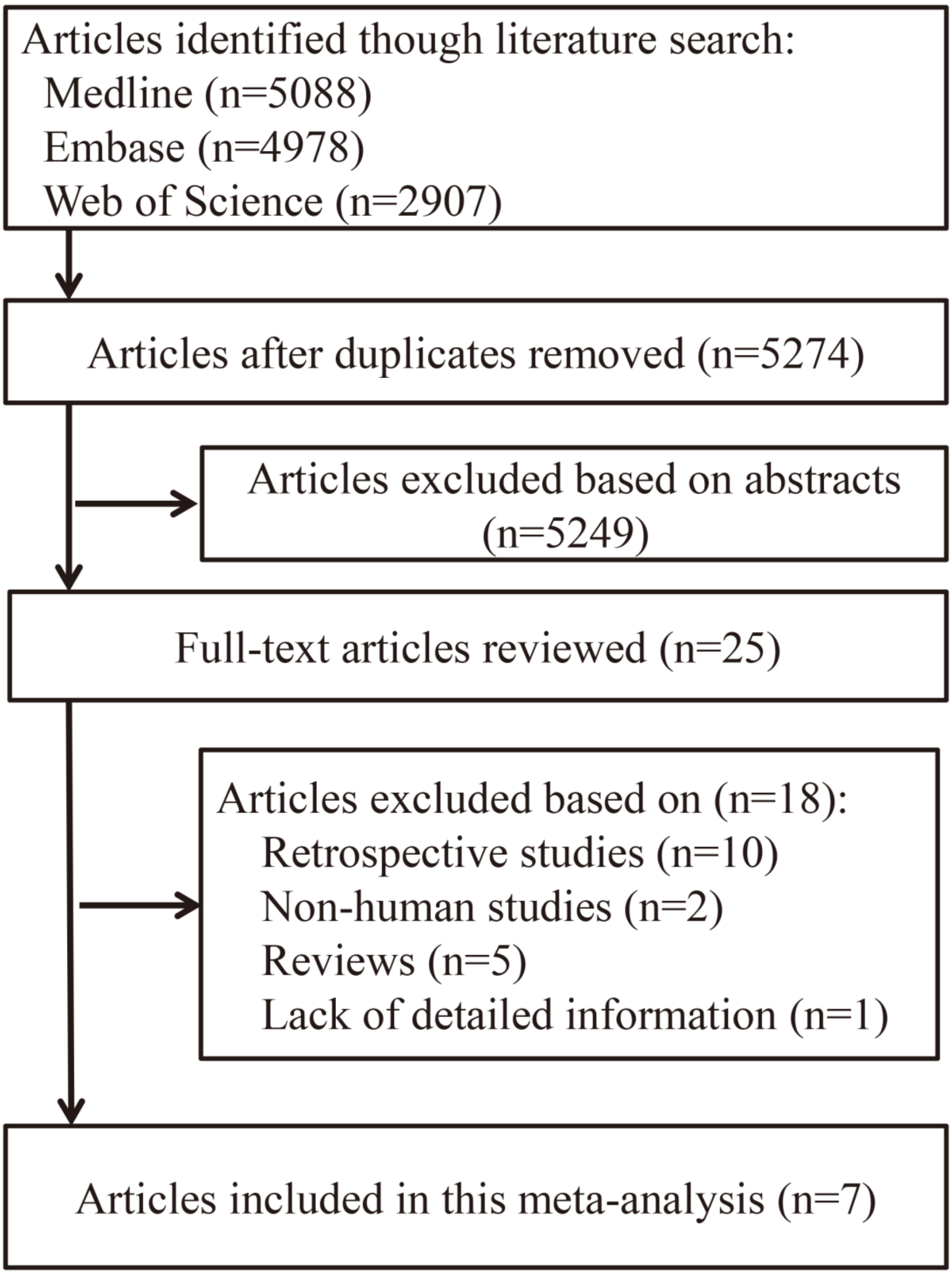

© 2018 Fang X et al. *JAMA Network Open.*

eFigure 2. Forest plot of PD risk for the highest versus the lowest categories of physical activity – excluding the first 4-10 years of follow-up

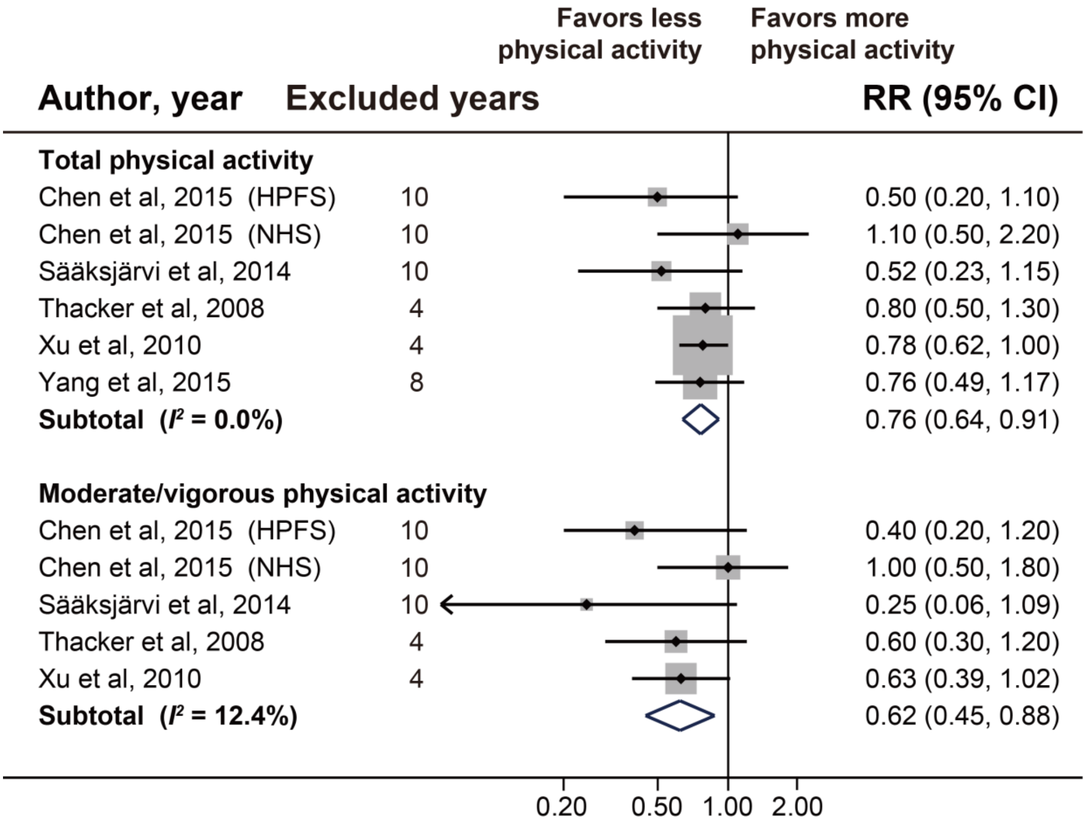

© 2018 Fang X et al. *JAMA Network Open.*
